# *Salix babylonica* L. as a Natural Anticoccidial Alternative in Growing Rabbits

**DOI:** 10.1155/2019/2107231

**Published:** 2019-08-29

**Authors:** Nallely Rivero-Perez, Jerelly L. Hernández-Alvarado, Benjamín Valladares-Carranza, Lucía Delgadillo-Ruiz, Deyanira Ojeda-Ramírez, Carolina G. Sosa-Gutiérrez, Ana L. Morales-Ubaldo, Vicente Vega-Sanchez, Adrian Zaragoza-Bastida

**Affiliations:** ^1^Área Académica de Medicina Veterinaria y Zootecnia, Instituto de Ciencias Agropecuaria, Universidad Autónoma del Estado de Hidalgo, Av. Universidad Km 1, Ex-Hda. de Aquetzalpa, C.P. 43600 Tulancingo, Hidalgo, Mexico; ^2^Centro de Investigación y Estudios Avanzados en Salud Animal, Facultad de Medicina Veterinaria y Zootecnia, Universidad Autónoma del Estado de México, Km 15.5 Carretera Panamericana Toluca-Atlacomulco, C.P. 50200 Toluca, Estado de México, Mexico; ^3^Unidad Académica de Ciencias Biológicas, Universidad Autónoma de Zacatecas, Zacatecas, Mexico

## Abstract

Coccidiosis in rabbit production is responsible for high morbidity, mortality, and economic losses. The use of natural antimicrobial substances in rabbits represents a promising way to improve their health and production. The aim of the present study was to assess the activity of *Salix babylonica* hydroalcoholic extract (SBHE) on the elimination of *Eimeria* spp. in rabbits. The phytochemical compounds and chemical composition of SBHE were determined. The cytotoxicity of SBHE was determined by a microwell assay using *Artemia salina*. Twenty-five New Zealand rabbits, 28 days old and 872 ± 171 g body weight (BW), were used in a completely randomized design. The rabbits were assigned to five groups of five rabbits, control group (A) received only basal diet (BD), B group received BD + 25 mg/kg BW of SBHE, C group received BD + 50 mg/kg BW of SBHE, D group received BD + 100 mg/kg BW of SBHE, and E group received BD + coccidiostat Baycox® (75 mg/kg body weight) for 28 days. Feces samples were collected at days 0, 7, 14, 21, and 28; morphological and morphometric identifications of *Eimeria* were carried out by the flotation technique and counting of oocysts by the McMaster technique. The rabbits were found naturally infected with *Eimeria* spp. The SBHE present phytochemicals with anticoccidial activity, and the cytotoxicity test indicate that this extract is nontoxic. This study demonstrates that oral administration of SBHE at 25 and 50 mg/kg BW reduced the release of oocysts per gram of feces. This effect was observed at day 14 and had the most significant effect at day 28 for both concentrations. The results indicate that SBHE could be a natural alternative for the control of coccidiosis in rabbit production.

## 1. Introduction

Rabbits are potential livestock commodities for alternative meat supplies as well as pets and laboratory animals. Rabbit meat contains high protein content, low fat, and cholesterol and is rich in calcium and phosphorus as well as high linoleic acid [1, 2]. Rabbit production is affected by different factors including viral, bacterial, and parasitic diseases. Coccidiosis in domestic rabbits (*Oryctolagus cuniculus*) is a parasitic disease caused by different species of genus *Eimeria* [3]. Eleven different species of *Eimeria* have been discovered in domestic rabbits; ten of these species colonize the intestinal tract and *Eimeria stiedae* infects the bile ducts [3, 4]. These species of *Eimeria* affect rabbits in different ways and intensities, according to their degree of pathogenicity, which can result in stunted growth and death, especially in young animals [3, 5].

The transmission of coccidiosis is due to the intake of food contaminated with feces containing the sporulated oocysts which develop within the digestive system of rabbits, where they reproduce causing lesions and are excreted again in stool to reinitiate the infection cycle [6]. Coccidiosis in rabbit production might be responsible for high morbidity, high mortality, and economic losses. Clinical signs of coccidiosis in rabbits are diarrhea, appetite loss, weight loss, dehydration, secondary sepsis, and death. However, it is common that rabbits present subclinical coccidiosis, characterized by reduced feed intake and higher feed conversion ratio [7, 8].

Coccidiosis is mainly controlled with in-feed anticoccidial drugs which have been proven effective in preventing coccidiosis. Current treatment of coccidiosis consists of sulfonamides, salinomycin, and robenidine, which may become toxic to young rabbits and pregnant females; however, few anticoccidial drugs exist for commercial use and those that are available are prescribed mainly for poultry [2, 9, 10]. Few effective products for the control and treatment of coccidiosis are available; *Eimeria* spp. can generate resistance against these anticoccidial drugs. Previous suggestions included an increase in the use and production of organic food, along with extracts of medicinal plants. There are reports that support the effect that plant extracts have on the elimination of oocysts of *Eimeria* spp. in rabbits, such as garlic (*Allium sativum*) and oregano (*Origanum vulgare*) extracts [11, 12]. However, data concerning the effects of medicinal plant extracts on the coccidiosis in rabbits are scarce. Thus, it is important to find alternatives for the treatment of coccidiosis.

*Salix babylonica* extract has been widely used in ruminants to improve health and productive parameters, with excellent results and without showing negative effects on animal health [13, 14]. The anthelmintic effect of *Salix babylonica* extract on gastrointestinal parasites in sheep and goats has been reported including those of the genus *Eimeria* [15, 16]. For these reasons, *Salix babylonica* hydroalcoholic extract may be an alternative treatment for coccidiosis in rabbit production.

Given the above, the aim of the present study was to assess the activity of *Salix babylonica* hydroalcoholic extract on *Eimeria* spp. elimination in rabbits.

## 2. Materials and Methods

### 2.1. Preparation of the Hydroalcoholic Extract

The leaves of *Salix babylonica* were harvested from Tulancingo de Bravo, State of Hidalgo, Mexico, during the months of June–August. For plant identification, the Herbarium of UNAM (Universidad Nacional Autonoma de Mexico) was consulted, and the vegetal specimen was identified as *Salix babylonica* L. (IBUNAM: MEXU: 9744).

The hydroalcoholic extract was prepared according to the methodology described by Rivero et al. [13] with some modifications. The fresh leaves were collected randomly from several young and mature trees, washed, and then dried at room temperature in the dark. The dried *Salix babylonica* L leaves (70 kg) were macerated using a hydroalcoholic solution of water: ethanol (30 : 70 v/v) in the proportion of 1 kg leaf per 8000 ml of solvent at room temperature for 48 h to obtain of the extract. The extract was filtered using gauze and Whatman filter paper (Whatman® 42). The solvent was eliminated using a rotary evaporator (Büchi R-300, Suiza) to obtain a semisolid extract, and this extract was lyophilized (LABCONCO®) and finally freeze-dried and stored at −4°C, until the phytochemical analysis and experimentation.

### 2.2. Qualitative Tests of Chemical Profile of Extract

The chemical profile of hydroalcoholic extract of *Salix babylonica* was made according to the procedure described by Bañuelos-Valenzuela et al. [17] with some modifications [9], and the chemical tests performed are shown in Table 1.

### 2.3. Chemical Composition of the *Salix babylonica* Hydroalcoholic Extract by Gas Chromatography

The chemical composition was determined by gas chromatograph (GC: Agilent Technologies series 6890N, USA), with polar column DB_WAXetr, at 250°C, 12.13 psi, and flow of 36.5 mL of He min^−1^. Conditions for the column were as follows: initial temperature 50°C, from 0 to 2 min, increase of 10 in 10°C up to 250°C, constant for 5 min, reduction to 50°C for 2 min with flow of 1.6 mL of He min^−1^ at 12.13 psi, and average velocity of 25 cm s^−1^. The flame ionization detectorionizing (FID) was used at 210°C with flow of 40 mL of H_2_ min^−1^ and a flow of 450 mL of air min^−1^. The standards (Sigma-Aldrich) were used in various concentrations (Table 2).

### 2.4. Brine Shrimp Lethality Test

The cytotoxicity of *Salix babylonica* hydroalcoholic extract was determined by a microwell assay using *Artemia salina* (brine shrimp), according with the procedure described by Solis et al. [18], with some modifications.

Brine shrimp eggs of *Artemia salina* were hatched in artificial sea water prepared from sea salt (38 gr/L) supplemented with 6 mg/L dried yeast and oxygenated with an aquarium pump. After 48 hours incubation in a warm room (29°C), nauplii were collected with a Pasteur pipette.

The hydroalcoholic extract was diluted with artificial seawater, serial dilutions were made in 96-well microplates, and the concentrations evaluated were 125 to 0.12 mg/mL. Each concentration was evaluated in triplicate. Tween® 80 (SIGMA P1754) was used a positive control. A suspension of nauplii containing 10–15 organisms (200 *μ*l) and a concentration to evaluate was added to each well. The covered plate was incubated at 29°C for 24 hours. Plates were examined under a stereo microscope, and the numbers of dead (nonmotile) nauplii in each well were counted.

The statistical analysis was carried out following the methodology described by Syahmi et al. [19]; based on the percentage of the mortality, the concentration that led to 50% lethality (LC50) of the nauplii was determined by using the graph of mean percentage mortality versus the log of concentration using Microsoft Excel, which also formulated the regression equations. These equations were later used to calculate LC50 values for the samples tested with consideration of value greater than 1.0 mg/mL, suggesting that the extract is nontoxic.

### 2.5. Animals and Management

The experiments were performed at the experimental farm of Academic Area of Veterinary Medicine and Zootechnics of the Autonomous University of Hidalgo State. Twenty-five New Zealand white rabbits, 28 days old (newly weaning), with 872 ± 171 gr of body weight were used, and the rabbits were naturally infected and belonged to a farm with a history of intestinal coccidiosis. Rabbits were kept in individual galvanized cages of size 80 cm wide × 50 cm long × 40 cm high and fed with a basal diet based on alfalfa hay, ground corn, canola paste, soybean paste, ground sorghum, molasses, soybean husk, wheat bran, and mineral premix. The diet contained 16% crude protein, 13.2% raw fiber, and 2.5 Mcal·kg^−1^ of metabolizable energy. The general conditions regarding hygiene and equipment were typical of this type of production, and the handling of animals was according to international bioethical standards and NOM-062-ZOO-1999 [20].

### 2.6. Experimental Design and Sampling

Before the beginning the experiment, the *Eimeria* species were identified using a flotation technique and the oocysts per gram of feces (OPG) were quantified with the technique of McMaster in order to confirm the natural infection by *Eimeria* spp.

The rabbits were randomly assigned to five groups with five rabbits (A, B, C, D, and E). The control group (A) received only basal diet (BD), B group received BD + 25 mg/kg body weight (BW) of *Salix babylonica* hydroalcoholic extract (SBHE) (22.5 mg in 100 gr of basal diet), C group received BD + 50 mg/kg BW of SBHE (45 mg in 100 gr of basal diet), D group received BD + 100 mg/kg BW of SBHE (90 mg in 100 gr of basal diet), E group received BD + coccidiostat Baycox® in the water for 7 consecutive days (75 mg/kg body weight). The rabbits consumed fresh water and food *ad libitum* during the 28 days of experimentation.

### 2.7. Evaluation of Anticoccidial Activity

Feces samples were collected with gauze placed under the cages and then placed in polyethylene bags and transferred to the laboratory at 4°C. This methodology was performed at 0, 7, 14, 21, and 28 days of experimentation. A morphological and morphometric identification of *Eimeria* species present in the experimental groups was made using the flotation technique [6, 21]. The oocyst count per gram of the feces (OPG) was quantified according to McMaster techniques with four repetitions per group [22]. The data were analyzed using PROC MIXED procedure of SAS (2002) with repeated measures; significant differences between treatment means and time were assessed using the Tukey procedure at *P* < 0.05 level.

## 3. Results

### 3.1. Chemical Composition of Hydroalcoholic Extract of *S. babylonica*

The SBHE exhibited unsaturation, phenolic oxidrils, coumarins, lactones, sterols, triterpenes, flavonoles, flavonoids, sesquiterpene lactone, saponins, and floratanins. Gas chromatograph determined that SBHE contained terpinene (0.3050 mg/mL), linalol (0.3901 mg/mL), thymol (0.5319 mg/mL), and carvacrol (0.4158 mg/mL), without detecting the presence of limonene (Table 3).

### 3.2. Brine Shrimp Lethality Test

**Figure 1:**
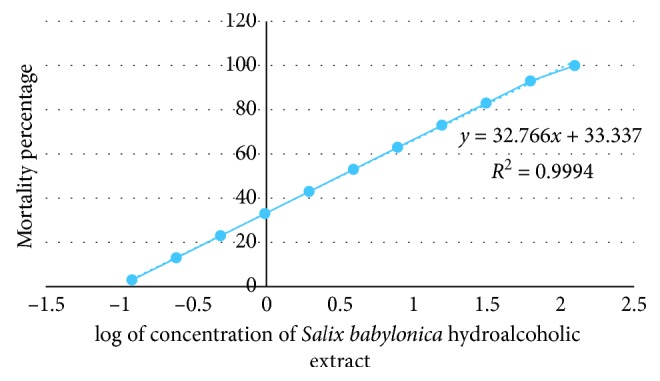
Brine shrimp lethality of *Salix babylonica* hydroalcoholic extract at 24 h.

The *Salix babylonica* hydroalcoholic extract showed positive results, indicating that the samples are biologically active. The extract resulting in LC50 values of less than 1 mg/mL is considered as significantly active which suggests that the SBHE, with LC50 values of 2.3 mg/mL at 24 hours, has a very low toxicity. Plotting of mortality percentage versus log of concentration of SBHE for all tests demonstrates an approximate linear correlation (Figure 1). Furthermore, there is a direct proportional relationship between the concentration of the extracts and the degree of lethality. This is shown by the fact the maximum mortalities occurred at a concentration of 125 mg/mL whilst a concentration of 0.12 mg/mL only caused minor mortalities.

### 3.3. Evaluation of Anticoccidial Activity

**Figure 2:**
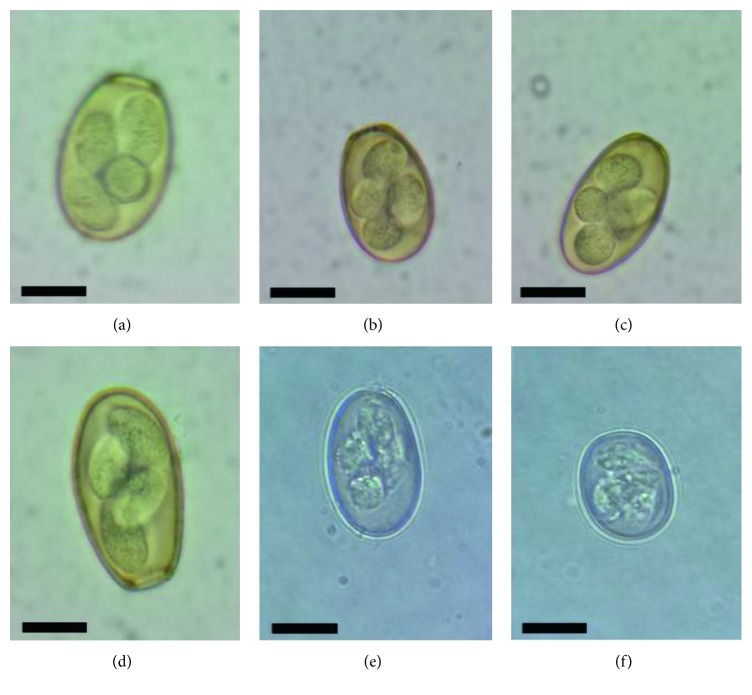
Light micrographs of oocysts of the six species of *Eimeria* collected from naturally infecting domestic rabbits. Scale bar = 10 *μ*m. (a) *E*. *stiedae*, (b) *E*. *magna*, (c) *E*. *coecicola*, (d) *E*. *media*, (e) *E*. *perforans*, and (f) *E*. *exigua*.

The multivariate analysis showed a significant interaction (*P* < 0.001) between time and treatment. Despite trying to form homogeneous groups, on day 0 of the experiment, there were statistical differences, treatments B, C, and D showed no significant differences (*P* > 0.05) as well as groups A and E as shown in the Table 4. Through morphological and morphometric examination, *E*. *stiedae*, *E*. *magna*, *E*. *coecicola*, *E*. *media*, *E*. *perforans*, and *E*. *exigua* were identified and all of them were distributed evenly in the experimental groups (Figure 2).

**Figure 3:**
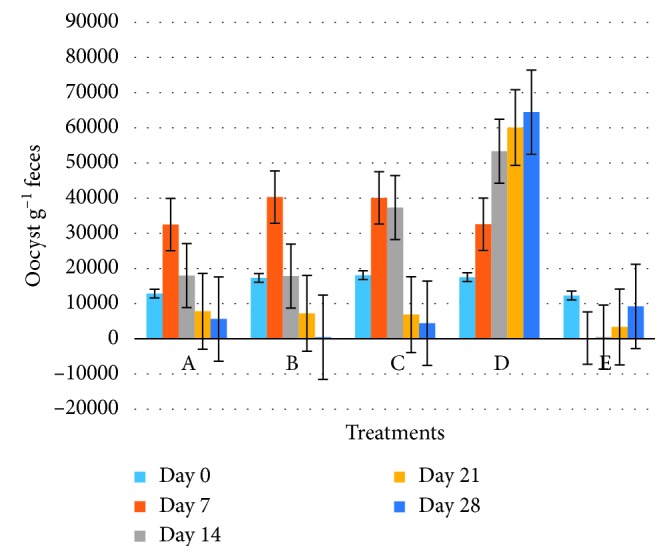
Efficacy of *Salix babylonica* hydroalcoholic extract on elimination of *Eimeria* spp. oocysts per group.

**Figure 4:**
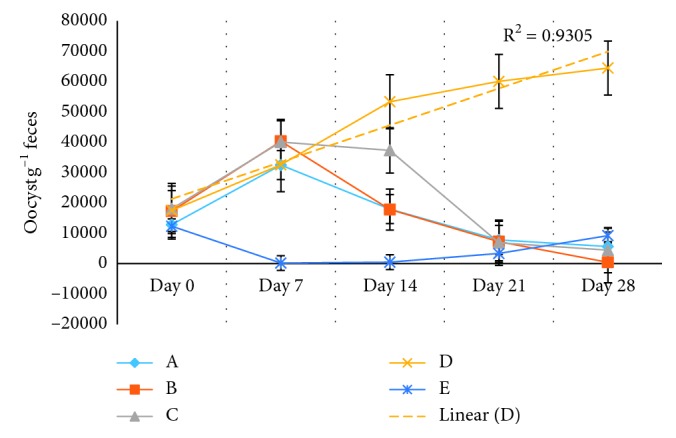
Efficacy of *Salix babylonica* hydroalcoholic extract on the elimination of *Eimeria* spp. oocysts the days 7, 14, 21, and 28.

At day 7, significant statistical differences (*P* < 0.001) were observed in the average of oocysts per gram of feces (OPG), between the treatment E (Baycox®) and treatments A, B, C, and D; in these groups, the OPG were increased, without significant statistical differences between them (*P* > 0.05) (Table 4, Figure 3), with the exception of the E group (Baycox®) in which the OPG decreased 98% (*P* < 0.001) with respect to day 0 (Table 5, Figure 4).

In the next sampling (day 14), significant statistical differences were observed (*P* < 0.001) in the average of OPG. In the A and C groups, the average OPG decreased but unlike values of day 0, showing significant statistical differences (*P* < 0.05) with respect to day 0 and unlike group B in which a decrease in the average OPG was determined, without presence of significant statistical differences (*P* > 0.05) between days 0 and 14. The D group did not present a reduction of OPG on day 14. Group E (Baycox®) did not exhibit significant statistical differences (*P* > 0.05) in the average of OPG with respect to day 7 and with a reduction of 96% in the average of OPG with respect to day 0 (Tables 4 and 5; Figures 3 and 4).

Samples observed on the day 21 had significant statistical differences (*P* < 0.001) in the average of OPG, in the A, B, and C groups; the average of OPG decreased to 37, 58, and 66%, respectively, with significant statistical differences between them (*P* < 0.001); group D did not present a reduction in the release of OPG. In group E, statistically significant differences (*P* < 0.001) were observed with respect to the percentage of OPG (73%) on days 7 (98%) and 14 (96%) (Table 5).

The final day of sampling (day 28) in the experimental groups indicated statistically significant differences (*P* < 0.001) in the average of OPG (Table 4). The A, B, and C groups presented a reduction of 56, 97, and 78%, respectively, with significant statistical differences between them (*P* < 0.05) (Table 4); the percentage of reduction of OPG of group B at day 28 did not show differences on days 7 and 14 of group E (Baycox®) (Tables 4 and 5). A linear tendency (*R* = 0.9305) was observed in group D in the release of oocysts per gram of feces (Figure 4). It is important to highlight that from day 21, the rabbits in the E group showed a reinfestation, because the average of OPG increased from 487.5 (day 14) to 3368.8 (day 21) until 9231.3 (day 28) (Table 4).

## 4. Discussion

Around the world, coccidiosis is a serious health and economic problem in rabbits, affecting mainly young rabbits after weaning [23]. The use of natural antimicrobial compounds is a promising way to improve health and commercial rabbit production. In the current work, the anticoccidial mechanism induced by *Salix babylonica* hydroalcoholic extract was not studied; however, previous studies demonstrated that phytochemical compounds of plants can suppress coccidiosis by intervention with the developmental stages of life cycle in *Eimeria* species [24]. The phytochemicals present in *Salix babylonica* hydroalcoholic extract with reports of the anticoccidial activity are coumarins, triterpenes, flavonoids, sesquiterpene lactone, saponins, terpinene, linalol, thymol, and carvacrol.

Studies conducted by Michels et al. [25] demonstrated the efficacy of coumestans (coumarin) from *Eclipta alba* against avian coccidiosis. Pop et al. [26] demonstrated the efficacy of artemisinin, a sesquiterpene lactone derived from *Artemisia annua*, against *Eimeria acervulina*, *Eimeria maxima*, and *Eimeria tenella* in poultry. De Pablos et al. [27] demonstrated the efficacy of maslinic acid (triterpene), from leaves and fruit of olive tree (*Olea europaea* L.), against *Eimeria tenella*. Ademola et al. [28] determined the activity of *Pleurotus ostreatus* extract (with saponins, flavonoids, anthraquinones, and alkaloids) against *Eimeria* spp. In avian in vivo studies realized by Remmal et al. [29], they demonstrated the efficacy of essential oil components (terpinene, linalol, thymol, and carvacrol) against chicken *Eimeria* oocysts. According to Muthamilselvan et al. [24], the flavonoids interfere with the life cycle of *Eimeria* species through oxidative stress and the saponins, terpinene, linalol, thymol, and carvacrol by destruction of oocysts and parasites.

The LC50 value of *Salix babylonica* hydroalcoholic was determined using the brine shrimp lethality test. According to Meyer et al. [30], extracts derived from natural products which have LC50 ≤ 1.0 mg/mL are known to possess toxic effects. In this study, the LC50 value of the crude extract is 2.3 mg/mL at 24 hours. These results prove that the *Salix babylonica* hydroalcoholic extracts are nontoxic.

The animals used in the present experiment were found naturally infected with multiple species of *Eimeria*; the morphological and morphometric examination allowed species identification, as follows: *E*. *stiedae*, *E*. *magna*, *E*. *coecicola*, *E*. *media*, *E*. *perforans*, and *E*. *exigua*; Heker et al. [7] identified ten species of *Eimeria* in Brazilian rabbit farms, *E*. *coecicola*, *E*. *flavescens*, *E*. *intestinalis*, *E*. *irresidua*, *E*. *magna*, *E*. *media*, *E*. *perforans*, *E*. *vejdovskyi*, *E*. *media*, and *E*. *stiedae*; on the other hand, García-Rubio et al. [31] identified *E*. *magna*, *E*. *media*, *and E*. *perforans* associated with enteric problems in rabbits from the State of Mexico, Mexico. The species identified in the present investigation are associated with high morbidity, mortality, and economic losses in commercial rabbit farms [23].

The results indicate that the oral administration of *Salix babylonica* hydroalcoholic extract has an effect on the release of OPG in rabbits naturally infected. The SBHE to 25 and 50 mg/kg of BW decreased the release of OPG; however, at the threshold, 100 mg had a negative effect and presented a linear increase in the release of OPG over time. Michels et al. [25] evaluated the efficacy of a food formulation with two different doses of coumestans from *Eclipta alba* (120 and 180 ppm) against avian coccidiosis; they determined that the food formulation containing the lower dose (120 ppm) showed a therapeutic effect on *Eimeria alba*, while the higher dose of coumestan proved to be inefficient as a therapeutic agent against avian coccidiosis, and severe destruction of the cecal lining was found in the intestinal tract of broilers fed with the product containing the higher dose (180 ppm).

In this same sense, Khalafalla et al. [32] evaluated the effects of curcumin (diferuloylmethane) on *Eimeria tenella* sporozoites in vitro and they determined that sporozoite infectivity was reduced at curcumin concentrations of 100 and 200 *μ*M by 41.6% and 72.8%, respectively, without observing negative effects of curcumin on Madin–Darby bovine kidney (MDBK) cells at these concentrations; however, curcumin at concentrations of 1800, 600, and 400 *μ*M was toxic to MDBK cells and affected cell proliferation. According to the studies conducted, the negative effect of SBHE may be due to the toxic effect that the extract has when the concentration of it is increased as shown in Figure 1.

The best results of *Salix babylonica* hydroalcoholic extract on the release to OPG were observed at 25 and 50 mg/kg of BW; however, statistical differences (*P* < 0.05) between the groups was determined; this effect was observed since day 14 and had the most favorable outcomes on day 28 for both concentrations. Cervantes-Valencia et al. [33] evaluated the hydroalcoholic extract of *Curcuma longa* in rabbits naturally infected with *Eimeria* spp. and determined that at doses of 25 and 40 mg/kg BW, the OPG of *Eimeria* spp. decreased within 24.2 and 80.1%, respectively, on day 28 [16], while the *Salix babylonica* hydroalcoholic extract decreased the release the OPG up to 78% (Group C) and 97% (group B) on day 28 of experiment.

The results of the present study showed that rabbits treated with *Salix babylonica* hydroalcoholic extract at 25 mg/kg of BW (Group B) had a reduction of OPG, starting 14 days after the ingestion of the extract, and on day 28, a reduction of 97% was observed without observing any statistical differences on days 7 and 14 of the group treated with Baycox® (Group E). This result coincides with that reported by Simonová et al. [34] who observed a reduction in the release of OPG in rabbits naturally infected with *Eimeria* spp., on day 21  of the administration of chamomile essential oil as well as that published by Indrasanti et al. [1] who observed the same result when administering garlic extract in infected rabbits with *Eimeria stiedai*.

The group of rabbits treated with Baycox® (Group E) exhibited a reduction of the OPG observed on days 7 (98%) and 14 (96%) as expected; nevertheless, from day 21, reinfection was evident, a situation that did not occur in groups B and C. This outcome coincides with the results published by Nosal et al. [11] who observed a reinfection in rabbits by *Eimeria* spp., five weeks after treatment with Baycox®.

## 5. Conclusions

*Salix babylonica* hydroalcoholic extract decreased the release of OPG in rabbits that were naturally infected with the *Eimeria* spp. This activity is due to its content of phytochemicals with anticoccidial properties such as coumarins, triterpenes, flavonoids, sesquiterpene lactone, saponins, terpinene, linalol, thymol, and carvacrol. The best results on the reduction of OPG were observed at 25 and 50 mg/kg of BW. This effect was observed since day 14 and had the most favorable effect on day 28 for both concentrations. *Salix babylonica* hydroalcoholic extract at 25 mg/kg of BW had a reduction of OPG of 97% on day 28 of the experimentation without observing any statistical differences on days 7 and 14 of the group treated with Baycox®. In fact, reinfection was observed in those groups on day 21. Results of the cytotoxicity test showed that *Salix babylonica* hydroalcoholic extract is nontoxic. *Salix babylonica* hydroalcoholic extract could be a natural alternative for the control of the coccidiosis in rabbit production.

## Figures and Tables

**Table 1:** Qualitative tests to determine the chemical profile of *Salix babylonica* hydroalcoholic extract.

Qualitative tests	Sample processing
Test with KMnO_4_ to detect unsaturation	2 mg of sample was resuspended in 1 mL of methanol, and KMnO_4_ at 2% was added drop by drop in water. The test was positive when there was discoloration or formation of brown precipitate.
Test with FeCl_3_ to detect phenolic oxydrils (vegetable tannins)	2 mg of sample was resuspended in 1 mL of water, and some drops of FeCl_3_ (III) at 12.5% in water were added. The test was positive when red, blue-violet, or green precipitate was formed.
Liebermann–Bouchard test to detect sterols and triterpenes	The reactive prepared by mixing 1 mL of acetic acid and 1 mL of chloroform, cooled to 0°C, with sulfuric acid added drop by drop until there was no chemical reaction, and added drop by drop to the sample. The test was positive when blue, green, red, or orange colors were developed during that time.
Salkowski test to detect sterols and triterpenes	2 mg of sample was dissolved in NaOH at 10%. The test was positive when it developed yellow coloration which was eliminated by acidulation of the mixture.
Test of coumarins	2 mg of sample was dissolved in 10% NaOH; if a yellow coloration appears, which disappears when the test is acidulate, the test is positive.
Baljet test to detect sesquiterpenlactones	2 mg of the extract was mixed with 3 or 4 drops of the mixture solution (acid picric and NaOH). The test was positive when the coloration changed from orange to dark red.
Test of H_2_SO_4_ to detect flavonoids	2 mg of the sample was dissolved in H_2_SO_4_. Yellow coloration indicated the presence of flavonoids, orange-maroon that of flavons, bluish-red that of chalcons, and reddish-purple that of quinones.
Shinoda test for flavonoids	2 mg of sample and 1 mL of ethanol were placed in a test tube, magnesium filings (0.5 g) and three drops of concentrated HCl were added. The presence of flavonoids was confirmed when orange, red, pink, and violet coloration developed.
Dragendorff test to detect alkanoids	Two or three drops of the A (bismuth nitrate and glacial acetic acid) and B (potassium iodate) reactive were added in 2 mg of sample. Orange to red coloring was considered positive.
Tannin test	1 mL of the sample and 20 mL of H_2_O were boiled in a test tube, and 3 drops of 0.1% FeCl_3_ were added. The positive test is considered if it appears green or blue-black color.
Phlorotannins test	1 mL of the sample was boiled with 20 mL of 1% HCl. The test is considered positive if there is a presence of a red precipitate.
Steroid test	2 mL of acetic acid was placed with 0.5 mL of the extract sample and 2 mL of H_2_SO_4_ in a test tube. The appearance of a blue-violet-green color is considered positive.
Sodium bicarbonate test	2 mg of the sample was dissolved in water, 3 drops of sulfuric acid were added, and 3 drops of a solution of sodium bicarbonate (10%) were added. The test is considered positive with the appearance of bubbles and its permanence for more than 1 minute indicating the presence of saponins.
Salkowski test for saponins	2 mg of sample was dissolved in 1 mL of chloroform, and 1 mL of sulfuric acid was added. The test is considered positive with the appearance of a red color.

**Table 2:** Concentrations of standards (mg·mL^−1^) to determine the chemical composition of *Salix babylonica* hydroalcoholic extract of by gas chromatograph.

	Terpenes (mg·ml^−1^)				
Standard	Thymol	Carvacrol	Linalol	Terpinene	Limonene
1	10.373	8.284	7.744	7.154	8.496
2	5.186	4.142	3.872	3.577	4.248
3	2.593	2.071	1.936	1.789	2.124
4	1.297	1.035	0.968	0.894	1.062
5	0.648	0.518	0.484	0.447	0.531
6	0.324	0.259	0.242	0.224	0.265

**Table 3:** Phytochemical compounds of *Salix babylonica* hydroalcoholic extract.

Qualitative tests of chemical profile		Chemical composition by gas chromatography	
Compounds	SBHE	Compounds	SBHE (mg/mL)
Unsaturation	+	Terpinene	0.3050
Phenolic oxidrils	+	Limonene	0
Coumarins	+	Linalol	0.3901
Lactones	+	Thymol	0.4721
Sterols	−	Carvacrol	0.3616
Triterpenes	+		
Flavonoles	−		
Flavonoids	+		
Chalcones	−		
Quinones	−		
Sesquiterpene lactone	−		
Saponins	+		
Aromaticity	−		
Triterpenes	−		
Tannins	−		
Floratanins	+		
Steroids	−		

Note: + = detected; − = not detected; SBHE = *Salix babylonica* hydroalcoholic extract.

**Table 4:** Efficacy of *Salix babylonica* hydroalcoholic extract on the elimination of *Eimeria* spp. oocysts in rabbits.

	Average of oocysts g^−1^ (time ± SD)				
Group	0	7	14	21	28
A	12868 ± 564^cB^	32496 ± 156^aB^	17962 ± 151^bC^	7825 ± 322^dB^	5631.3 ± 177^eC^
B	17318 ± 597^bA^	40318 ± 108^aA^	17834 ± 100^bC^	7231 ± 307^cB^	443.8 ± 16^dE^^*∗*^
C	18093 ± 371^cA^	40062 ± 161^aA^	37312 ± 338^bB^	6875 ± 322^dB^	4437 ± 161^eD^
D	17525 ± 277^eA^	32562 ± 161^dB^	53343 ± 363^bA^	60062 ± 161^cA^	64450 ± 322^aA^
E	12325 ± 322^aB^	200.0 ± 10^dC^^*∗*^	487.5 ± 32^dD^^*∗*^	3368.8 ± 48^cC^	9231.3 ± 306^bB^

^abc^Different letters within a row indicate significant statistical differences in the time (*P* < 0.05). ^ABC^Different letters within a column indicate significant statistical differences in the treatment (*P* < 0.05). ^*∗*^No statistical differences between treatments over time (*P* > 0.05).

**Table 5:** Effect of *Salix babylonica* hydroalcoholic extract on the reduction percentage of *Eimeria* spp. oocysts in rabbits with respect to day 0 of the experiment.

	Day			
Group	7 (%)	14 (%)	21 (%)	28 (%)
A	0	0	37 ± 5.29^d^	56 ± 3.31^c^
B	0	0	58 ± 0.331^c^	97 ± 0.1^a^*∗*^^
C	0	0	66 ± 2.24^b^	78 ± 1.2^b^
D	0	0	0	0
E	98 ± 0.04^A^*∗*^^	96 ± 0.36^A^*∗*^^	73 ± 0.08^aB^	25 ± 4.45^dC^

^abc^Different letters within a column indicate significant statistical differences (*P* < 0.05). ^ABC^Different letters within a row indicate significant statistical differences (*P* < 0.05). ^*∗*^No statistical differences between treatments over time (*P* < 0.05).

## Data Availability

The data used to support the findings of this study are available from the corresponding author upon request.
